# Physical violence against health staff by mentally ill patients at a psychiatric hospital in Botswana

**DOI:** 10.1186/s12913-018-3187-6

**Published:** 2018-05-11

**Authors:** Anthony A. Olashore, Oluyemi O. Akanni, Radiance M. Ogundipe

**Affiliations:** 1 Department of Psychiatry, Faculty of Medicine, University of Botswana, Gaborone, Botswana; 2 Clinical Services, Federal Neuropsychiatric Hospital, Benin City, Edo State Nigeria; 3 Department of Family Medicine, Faculty of Medicine, University of Botswana, Gaborone, Botswana

**Keywords:** Botswana, Health Staff, Mentally ill, Physical violence, Psychiatric hospital

## Abstract

**Background:**

Workplace violence is worrisome in the mental health sector. Little is understood about it in sub-Saharan Africa. Consequently, we decided to investigate the prevalence, related factors, and the available sources of support for the victims of workplace violence in a mental referral hospital in Botswana.

**Methods:**

We conducted a cross-sectional retrospective survey of 201 mental health staff (MHS) of Sbrana Psychiatric Hospital, Botswana. We used a self-administered questionnaire to obtain information on socio-demographics and various aspects of work-related violence and available source of supports. We also used Andrew and Withey Job Satisfaction Questionnaire to assess the workers’ level of job satisfaction.

**Results:**

One hundred and seventy-nine questionnaires out of the two hundred and one returned were analyzed. One hundred and twenty-five (69.8%) of the respondents reported a lifetime experience of physical violence, while 44.1% experienced the same during the previous 12 months. Nursing services (χ2 = 29.95, *p* < 0.01) and long duration of service (χ2 = 29.95, *p* < 0.01) were associated with lifetime encounter of physical violence. Those who reported a physical assault had a higher level of job dissatisfaction than staff who never experienced violence (*t* = − 3.07, *p* = 0.02).

**Conclusions:**

The rate of physical violence among mental health workers in Botswana is comparably high, and nurses are the most exposed members of staff. Protocol development and periodic training on violence prevention are hence recommended, especially for the most exposed members of staff.

## Background

Workplace violence is global and worrisome especially in the health sector, where it is mostly endured, under-reported, or often neglected [1, 2]. Health workers commonly accept it as an occupational hazard and a risk considered as a consequence of health care delivery [3, 4]. Most of the violence at workplace comprises of verbal threats, assault such as stalking, physical assault, sexual harassment or rape [3]. These acts may be perpetrated by professional colleagues, other hospital employees, clients or their relatives in a hospital setting [5].

Prevalence of violence varies with the job description of the care provider, type of training, duration of employment, type and severity of client’s disorder. Nurses, doctors, workers at the emergency and psychiatric services are at higher risk of any form of violence than other health care staff [3, 4, 6, 7]. Other factors such as female predominated staff, younger age of staff, and levels of work experience in a psychiatric facility are likewise associated with the experience of patients caused violence [8].

Violence caused by mentally ill patients against mental health care providers is disturbing, and the prevalence is significantly high [6, 9, 10]. For example, in a study conducted in Europe, about 70% reported being physically attacked by patients in the past 12 months, and half of the reported cases occurred in a psychiatric facility [9]. In America, 40% of psychiatrists reported being assaulted physically [3]. Another study carried out in a psychiatric hospital in West Africa revealed a similar trend as those from developed countries [4]. In the study, about half (49.5%) of mental health care providers had experienced physical assault by patients at least once within their employment period in the psychiatric facility, and 33.7% had been physically assaulted in the past 12 months [4].

Despite the massive burden of diseases in sub-Saharan Africa, there is an acute shortage of specialists particularly in the field of mental health [11]. In Botswana for instance, only one mental referral hospital caters for all the mental health referrals from other levels of health care, including general tertiary hospitals and as such, there is less than one psychiatrist per 100,000 population [12]. This situation puts much pressure on this institution and the staff and heightens the risk of occupational hazard. There is a perceived disparity by the media in Botswana regarding reportage of violent treatment of mentally ill patients compared to violence against health workers [13]. Moreover, to the best of our knowledge, most studies from Botswana are patient-focused, and none is focused on the welfare of the care providers. Work-related violence against care providers will continue in the absence of data to assist in the formulation of necessary preventive measures. We, therefore, decided to provide this, by investigating the prevalence of violence, related factors and the available sources of support for the victims of workplace violence. We believe this would assist in raising awareness of this hazard among all concerned stakeholders, and lead to the development of protocols to combat it.

## Methods

This is a cross-sectional retrospective study conducted at the Sbrana Psychiatric Hospital (SPH), Lobatse. The hospital is a 300-bed facility which provides in-patient and out-patient care, as well as emergency services to mentally ill persons in Botswana. Like most other psychiatric settings [4], the wards are divided into acute wards for those who are critically disturbed, and rehabilitation wards for patients who have either improved mentally or are less disturbed. Other wards include the forensic wards and psycho-geriatric wards, for both males and females. The hospital also has many other units such as the laboratory, occupational therapy, psychology, and social services.

A previous definition of physical violence was operationally used in this study [4, 14]. It defined violence as any act of physical aggression during which the patient deliberately and forcefully hits a care provider with any part of his or her body or with an object, with the intent of inflicting either physical or psychological pain or injury to the target. Hence, physical violence included kicking, pushing, shaking, biting, strangle-holding with attendant injury or discomfort, blushing or hurting of the skin, raping or attempting rape with apparent damage to the apparel, excluding mere threats or verbal violence.

The measuring instrument had three sections. Section one was designed to inquire about the socio-demographic characteristics of the mental health care providers. The second section enquired about physical violence, and similar questions used by previous researchers [4, 14] were adopted here, though some modifications were made to fit the objectives of the study. It contained questions about the frequency and nature of physical violence, history, location and the circumstance in which it occurred. The last section contained the Andrew and Withey Job Satisfaction Questionnaire, a uni-dimensional questionnaire that measures global job satisfaction [15]. It was used to assess job satisfaction. It consists of five items, with responses given on a seven-point Likert scale ranging from delighted (1) to terrible (7), and can be used for all population including hospital personnel. The convergent validity and internal consistency are 0.78 and 0.81 respectively and are satisfactory.

Clearance to embark on the study was obtained from the Ethics and Research Committee of the University of Botswana, the Ministry of Health, and the management of SPH. Thereafter, the questionnaires were administered to every consenting mental health staff (MHS) of the hospital. The study population consisted of psychiatrists, medical officers, psychiatric nurses, general nurses, psychologists, occupational therapist, laboratory scientists, social workers, auxiliary staff, laboratory technicians and record officers. The period of the study lasted for 4 months (1st of August to 30th of November, 2016) to allow those on shift duty and those on leave to be part of the study.

Statistical analysis was done using Statistical Package for Social Sciences for window-version 19 (SPSS-19). Descriptive statistics such as frequencies, means, and standard deviations were used to describe the pattern and prevalence of physical violence. Other statistical computations such as independent t-tests were used to analyze the relationship between physical violence and job satisfaction, while chi-square tests were used to determine the association between physical violence and other variables such as profession, religion, marital status and age group. The level of significance was set at *P* < 0.05.

A post hoc analysis of the power of the study was carried out using the G*power 3.1.9.8 software. At a medium effect size of 0.5, it was found to be 86 and 91% for 12 months and lifetime violence respectively, when compared with those who did not report violence.

## Results

Two hundred and ten health staff were available during the study period, but nine of them refused to participate. One hundred and seventy-nine (85.2%) responses were analyzed out of the remaining 201 questionnaires that were returned. The remaining questionnaires were excluded from analysis due to incomplete responses on the variables of interest. Table 1 describes the characteristics of the study population. More females (59.8%) participated in the study than males. The median age of the respondents was 32 years, and the majority were 32 years and below (55%). Most of the respondents were unmarried (60.6%) and practiced the Christian religion (95.4%). A high percentage (78.1%) of the respondents worked in the nursing services, and more than half of the staff had only worked for 4 years and less.

**Table 1:** Socio-demographic characteristics of the participants

Characteristic	No of participants (%)
Gender	179(100%)
Male	72(40.2%)
Female	107(59.8%)
Age(years)	171^a^(100%)
< =32	94(55.0%)
> 32	77(45.0%)
Marital status	178^a^(100%)
Single	108(60.6%)
Married	68(38.2%)
Divorced/Widowed	1(0.6%)
Widowed	1(0.6%)
Religion	175^a^(100%)
Christianity	167(95.4%)
Islam	1(0.6%)
African traditional religion	3(1.7%)
Others	4(2.3%)
Profession	178^a^(100%)
Psychiatrist	4(2.2%)
Medical officer	6(3.4%)
Psychiatric nurse	31(17.4%)
General nurse	108(60.7%)
Student nurse	2(1.1%)
Psychologist	3(1.7%)
Social worker	7(3.9%)
Occupational therapist	1(0.6%)
Pharmacist	5(2.8%)
Record officer	5(2.8%)
Auxiliary staff	4(2.2%)
Lab technician	2(1.1%)
Income per month	179 (100%)
= < 5000	13(7.3%)
6-10,000	56(31.3%)
11-20,000	95(53.1)
> 20,000	15(8.4%)
Duration of practice (years)	176^a^ (100%)
< =4	89(50.6%)
> 4	87 (49.4%)
Lifetime attack	179(100%)
No	54(30.2%)
Yes	125 (69.8%)
Past 12 months attack	179(100%)
No	100(55.9%)
Yes	79 (44.1%)

Median age is 32 years

Median duration of practice is 4 years

12 months prevalence of violence is 44.1%

Lifetime prevalence of violence is 69.8%

^a^Figure did not add up to 179 because of missing data

**Fig. 1:**
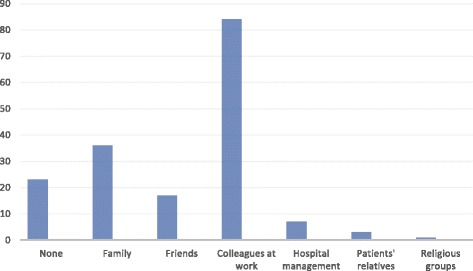
Common sources of support for physically assaulted mental health care staff

Of those who participated in the study, a total of 125 (69.8%) reported a lifetime experience of physical violence by patients, while only 79 (44.1%) reported being attacked in the last 12 months (Table 1). The most prevalent type of physical violence was being hit, and it accounted for 41.1%. This was followed by kicking (21.8%), pushing (20.2%) and shaking (12.1%). The highest number of attacks took place in the acute wards (58.1%); others occurred in the wards (22%) such as the rehabilitation, observation, psycho-geriatric wards and day hospital. The most frequent events preceding attacks occurred while attempting to calm patients (33.3%) and some occurred when patients were unprovoked (16.9%) (Table 2). More than half (61.5%) of the respondents believed these acts could be prevented while fewer (27%) believed it could be predicted. Twenty-seven percentage (48) of those who were attacked sustained physical injury, but only 18.5% (33) of them required medical treatment. Over two-thirds (72.6%) of them had emotional support, 11 (8.9%) had other forms of support such as financial, medical and time off from work, while 23 (18.5%) had none. The most common source of support was from other colleagues at work, followed by family and friends (Fig. 1).

**Table 2:** Frequency of type, location, and antecedent events to the attacks

Type of attack	N (%)	Location	N (%)	Antecedent events	N (%)
Hit	51(41.1)	Acute wards	72(58.1)	Attempting to calm patient	42(33.9)
Shaken	15(12.1)	OPD/Clinic	5(4.0)	Restraining aggressive patient	15(12.1)
Struck with an object	11(8.9)	Office	2(1.6)	Talking with patient	20(16.1)
Kicked	27(21.8)	Forensic ward	9(7.3)	Giving instruction to the patient	11(8.9)
Pushed	25(20.2)	Other wards^c^	28(22.6)	Unprovoked	21(16.9)
Attempted strangling	11(8.9)	Other places^d^	7(5.6)	Serving medications	1(0.8)
Bitten	12(9.7)	Can’t remember	1(0.8)	Others^b^	12(9.7)
Attempted rape	1(0.8)			Can’t remember	2(1.6)
Stabbed with knife	__				
Spat on	1(0.8)				
Others^a^	15(8.4)				
Can’t remember	4(3.3)				
Total	173(100)		124(100)		124(100)

^a^Others: scratched with nails, splashed water, or poured food on, spat on, etc

^b^Others: sample taking, serving food, feeding patients, bathing patients, etc

^c^Other wards: rehabilitation wards, observation words, psychogeriatric wards and day hospital

^d^Other places, corridors, bathrooms, receptions

Nurses were significantly more exposed to physical violence in their lifetime (χ2 = 29.95, *p* < 0.01) and in the past 12 months (χ^2^ = 26.91, *p* < 0.01) when compared to every other healthcare provider. Similarly, those who had worked for more than 4 years (χ^2^ = 29.95, *p* < 0.01) were more exposed to lifetime physical violence than those who had not (Table 3).

**Table 3:** Association of 12 months and lifetime violence with socio-demographic variables

Variables	LIFETIME VIOLENCE				12 MONTHS VIOLENCE			
	No (%)	Yes (%)	**χ** ^**2**^	*P*	No (%)	Yes (%)	**χ** ^**2**^	*p*
Gender								
Male	22(30.6)	50(69.4)	0.00	0.93	35(48.6)	37(51.4)	2.57	0.11
Female	32(29.9)	75(70.1)			65(60.7)	42(39.3)		
Age (years)								
< =32	30(31.9)	64(68.1)	0.72	0.40	52(55.3)	42(44.7)	0.01	0.95
> 32	20(26.0)	57(74.0)			43(55.8)	34(44.2)		
Religion								
Christianity	51(30.5)	116(69.5)	1.19	0.28	94(56.3)	73(43.7)	1.22	0.73
Others	1(12.5)	7(87.5)			4(50.0)	4(50.0)		
Profession								
Doctors	6(60.0)	4(40.0)	22.7	< 0.01	10(100.0)	–	26.2	< 0.01
Nurses	29(20.6)	112(79.4)			65(46.1)	76(53.9)		
Other clinical	11(68.8)	5(31.2)			13(81.2)	3(18.8)		
Non clinical	7(63.6)	4(36.4)			11(100.0)	–		
Marital Status								
Married	18(26.5)	50(73.5)	0.58	0.45	40(58.8)	28(41.2)	0.46	0.50
Unmarried	35(31.8)	75(68.2)			59(53.6)	51(46.4)		
Years of practice								
< =4	42(47.2)	47(52.8)	29.9	< 0.01	55(61.8)	34(38.2)	2.73	0.10
> 4	11(12.6)	76(87.4)			43(49.4)	44(50.6)		

Staff who had experienced violence in their lifetime reported significantly higher mean scores on the Andrew and Withey Job Satisfaction Questionnaire when compared to those who had not (*t* = − 3.07, *p* = 0.02). There was no association between previous year experience of violence and job satisfaction (Table 4).

**Table 4:** Comparison between the experience of violence (Lifetime and 12 months) with the Job satisfaction scores

Violence	Mean	SD	t	P
Lifetime			−3.07	0.02
No	17.75	5.41		
Yes	20.42	5.23		
12 months			−1.82	0.07
No	18.97	5.60		
Yes	20.44	5.07		

df = 176

## Discussion

The current study reveals that physical violence against mental health workers exists in Botswana like in other parts of the world [4, 6, 9, 10]. The lifetime and 12 months prevalence rates obtained in this study are respectively higher than the rates of 49.5 and 30.6% previously reported by Ukpong et al. [4]. The disparity may be attributed to the fact that, unlike the survey by Ukpong et al. which was restricted to professional MHS, expectedly more competent in handling potentially aggressive patients, the present study included other hospital workers that are not suitably trained for such and may be more at risk of experiencing violence. The high prevalence of violence in this study underscores the need to take critical actions in curtailing the occurrences which have become a public health threat. Various ways have been proposed, and these include raising awareness on the likelihood of the event, frequent incidence reporting and reviewing, adequate staffing, the use of different methods of restraint, and regular training in early identification of potentially violent patient [3, 16]. It has also been suggested that such training should incorporate attitudinal change regarding the prevention and particularly, the prediction of violence [3, 16]. This is necessary because, the current study has further shown that, about two-fifths (38.5%) of the respondents believed these acts could not be prevented while 73% believed they could not be predicted. Such faulty beliefs may lead to apathy toward potential threats. Apathy toward potential threats and infrequent incidence reporting may create a neighborhood conducive to more violence or increase the vulnerability of MHS to more dangerous physical attacks.

Most of the attacks reported by the participants of this study occurred in the acute ward. Our finding does not replicate findings from other studies, which have reported a higher prevalence of attacks in the emergency rooms compared to other units in the hospital [3, 16]. This finding is not surprising because, at SPH, there is no emergency unit; patients who come into the hospital are quickly triaged at the out-patient unit. Those who are very disturbed and have higher tendencies to be violent are transferred to the acute wards. Thus, the acute ward in SPH shares similarities with emergency rooms of other hospitals and that could be a risk for violence incidence. Also, this part of the hospital, like in most other centers [8] especially those in resource-constrained countries [4], is often overcrowded and understaffed. This may further explain the high occurrence of violence in this unit.

The results of this study support earlier investigations that nurses are significantly more at risk than other healthcare providers in their lifetime and over the past 12 months to experience violence and aggression perpetrated by patients. Studies have established that, apart from the fact that nurses spend more time with patients, and set rules and limits on the permissible type of behavior, they are, of all members of the health team, the closest to the patients [10, 17]. This finding is useful in guiding the development of protocols in violence prevention programs, as emphasis may need to be directed at this group of highly exposed professionals. Similarly, staff in the acute ward would benefit from this kind of targeted intervention.

Longer duration of service in the psychiatric hospital was found to be associated with violence in the present study, which is similar to the study by Ukpong and colleagues. The authors compared physical assaults by psychiatric patients against the staff of two psychiatric hospitals and found that the staff in the hospital where physical assaults were higher had long years of employment [4]. Longer duration of service in this health facility perhaps may only translate to more exposure to violence. In other words, this finding suggests that the number of years spent in service alone does not correlate with a wealth of experience in escaping violence, and frequent sessions of training and retraining of staff may be necessary.

In the present study, there was no difference in gender or the two age-categories studied of those who reported being attacked, both in their period of employment in the hospital and in the past 12 months. There have been conflicting reports on factors that influence the risk of violence against health care providers. Some authors believe that female staff, and older staff are associated with higher risk of violence in health care services [8, 18], while some have contrary reports [6]. For example, an Arabian study found a positive relationship between male staff and violence, unlike what has been reported in earlier studies [6, 8]. This disparity may be related to the cultural practice in the region which gives extra respect to the female [6]. The disparity in gender association with violence is partly a reflection of cultural influence or other stronger but unexplored factors which could be investigated in future studies.

Our study also revealed that 27% of the respondents experienced physical injury following physical attacks by patients. The adverse effects of violence against health care providers are enormous and have been widely reported [9, 10]. Physical impairment is one of the negative consequences of violence, which can be in the form of abrasions, lacerations, sprains, fractures or even loss of body parts sometimes leading to permanent disability. Although we did not explore the types of physical injuries sustained by the respondents in our study, about 18% sustained injuries which required medical treatment, and this is comparable to the rates observed in previous studies [6, 9]. Other common reactions to physical violence as reported by victims in hospital studies are emotional disturbances such as fear, anxiety, anger, depression, irritability, loss of confidence and burnout [6, 9, 10]. These emotional disturbances, though not explored, may explain the significant association between violence and poor job satisfaction found in this study. A similar association has been formerly reported among clinical staff [1, 19].

It is notable to find that respondents reported receiving little support from the management (i.e., employer) and the relatives of the perpetrators. The consequences of physical attacks or violence on care providers, lack of support from the management and opportunity to seek redress may have an untoward effect on productivity and service delivery if not adequately addressed. Therefore, in addition to reporting assaults, policies on seeking redress should be put in place. Adopting incident reporting procedures and frequent reviews will assist in understanding the causes of violence in this environment and formulating methods of prevention.

Lastly, there should be frequent training and adequate staffing with more mental health professionals, especially in those wards where potentially violent or very disturbed patients are admitted.

## Limitations

The study is subjected to recall bias because the variable of interest, physical violence, occurred in the past. The cross-sectional nature of the study limits the ability to determine the direction of causality between associations. Generalization of our findings which was conducted in a mental health care facility to other general hospitals in the country which attend to less severe psychiatric cases is limited.

## Conclusion

A significantly high percentage of staff at SPH had experienced physical violence in their lifetime. Physical violence was significantly associated with nursing staff, more years of practice and job dissatisfaction in the participants’ lifetime. Protocol development and periodic training on violence prevention are therefore recommended. More considerable attention should be explicitly given to nurses and generally to staff in the acute ward who are often recipients of violence.

## Abbreviations

MHS: mental health staff
SPH: Sbrana Psychiatric Hospital
